# A preliminary report on the contact-independent antagonism of *Pseudogymnoascus destructans* by *Rhodococcus rhodochrous* strain DAP96253

**DOI:** 10.1186/s12866-014-0246-y

**Published:** 2014-09-26

**Authors:** Christopher T Cornelison, M Kevin Keel, Kyle T Gabriel, Courtney K Barlament, Trudy A Tucker, George E Pierce, Sidney A Crow

**Affiliations:** Applied and Environmental Microbiology, Georgia State University, 161 Jesse Hill Jr. Dr, Atlanta, GA USA; Department of Pathology Microbiology & Immunology, University of California Davis, One Shields Avenue, Davis, CA USA

**Keywords:** *Pseudogymnoascus destructans*, Mycelia, Conidia, *Rhodococcus rhodochrous*, White-Nose Syndrome, Biocontrol

## Abstract

**Background:**

The recently-identified causative agent of White-Nose Syndrome (WNS), *Pseudogymnoascus destructans*, has been responsible for the mortality of an estimated 5.5 million North American bats since its emergence in 2006. A primary focus of the National Response Plan, established by multiple state, federal and tribal agencies in 2011, was the identification of biological control options for WNS. In an effort to identify potential biological control options for WNS, multiply induced cells of *Rhodococcus rhodochrous* strain DAP96253 was screened for anti-*P. destructans* activity.

**Results:**

Conidia and mycelial plugs of *P. destructans* were exposed to induced *R. rhodochrous* in a closed air-space at 15°C, 7°C and 4°C and were evaluated for contact-independent inhibition of conidia germination and mycelial extension with positive results. Additionally, *in situ* application methods for induced *R. rhodochrous,* such as fixed-cell catalyst and fermentation cell-paste in non-growth conditions, were screened with positive results. *R. rhodochrous* was assayed for *ex vivo* activity via exposure to bat tissue explants inoculated with *P. destructans* conidia. Induced *R. rhodochrous* completely inhibited growth from conidia at 15°C and had a strong fungistatic effect at 4°C. Induced *R. rhodochrous* inhibited *P. destructans* growth from conidia when cultured in a shared air-space with bat tissue explants inoculated with *P. destructans* conidia.

**Conclusion:**

The identification of inducible biological agents with contact-independent anti- *P. destructans* activity is a major milestone in the development of viable biological control options for *in situ* application and provides the first example of contact-independent antagonism of this devastating wildlife pathogen.

## Background

The rapid spread and high mortality rates associated with white-nose syndrome (WNS) make the development of *in situ* treatment options for the causative agent, *Pseudogymnoascus destructans* [1,2], a significant objective for wildlife management agencies. Accordingly, the development of biologically-derived treatment options may have advantages over chemical or physical treatments, since classic examples of chemical and physical treatments in karst environments are now a cautionary tale [3]. To this end, “A National Plan for Assisting States, Federal Agencies, and Tribes in Managing White-Nose Syndrome in Bats” [4] was released in May, 2011. In this plan, significant focus was placed on the identification and development of biological control options for WNS.

*Rhodococcus rhodochrous* strain DAP 96253 is a ubiquitous, soil-associated, Gram-positive bacterium with tremendous metabolic and physiological diversity [5-9]. *Rhodococcus rhodochrous* has been used extensively in bioremediation as well as in the production of nitrile-containing compounds [5-7] and it has demonstrated delayed fruit ripening activity with climacteric fruits and vegetables [8]. Several enzymes have been shown to have increased activity and prevalence in bacteria induced to delay fruit ripening and these enzymes may play a role in the observed antifungal activity [8]. Initial investigation of the potential antagonism of *P. destructans* by *R. rhodochrous* indicated that, when induced under the protocol outlined in US patents 7,531,343, and 7,531,344 [10,11], *R. rhodochrous* strain DAP 96253 demonstrated significant contact-independent antagonism of *P. destructans in vitro*. As a result, the principal objective of this was evaluation of *R. rhodochrous* induced with urea for potential *in situ* application as a biological control agent for *P. destructans*.

In addition to the strong evidence established via *in vitro* analysis of the observed antagonism, the evaluation of the efficacy of induced *R. rhodochrous* was pursued in order to establish *in vivo* efficacy at preventing fungal invasion of bat tissue. This goal was accomplished using a bat-skin explant assay. The evaluation of induced *R. rhodochrous* to prevent or reduce the infective potential of *P. destructans* conidia was demonstrated by the inhibition of *P. destructans* growth on living bat tissue. This is the first example of antifungal efficacy on living bat skin for any biological control agent of WNS and represents a major milestone in this effort.

In order to optimize biocontrol efficacy and reduce potential cross-contamination of karst environments, various whole- and fixed-cell applications were investigated. The evaluation of various application methods of induced cells of *R. rhodochrous* for potential *in situ* application, including whole-cell application, non-growth fermentation cell-paste, and fixed-cell catalyst [8,12,13], were conducted. Non-growth fermentation cell-paste demonstrated persistent inhibitory activity and represents the most promising application method evaluated. The associated cell-paste activity is a significant development as it represents multiple hallmarks of ideal biocontrol agents.

## Methods

### Culture acquisition and maintenance

All *P. destructans* isolates used in the project were acquired from the WNS diagnostic lab at The University of Georgia Southeastern Cooperative Wildlife Disease Study (UGA SCWDS). Initial investigations have shown very low genetic and physiological variability amongst *P. destructans* isolates [14]. Accordingly, all assays were conducted with a small isolate sample size (n ≤ 3). *P. destructans* cultures were maintained on Sabouraud Dextrose Agar (SDA, Difco) or in Sabouraud Dextrose Broth (SDB, Difco) at 4°C, 7°C, or 15°C depending on anticipated usage. *P. destructans* conidia were harvested from fungal lawns on SDA plates by adding 10 ml of conidia harvesting solution (CHS; 0.05% Tween 80, 0.9% NaCl) to the surface of the plate and gently scrapping with a sterile loop to dislodge conidia. The resulting solution was filtered through glass wool and centrifuged at 5000 rpm for 10 minutes. The resulting supernatant was removed and the spore pellet washed with 5 mL of sterile phosphate buffered saline (PBS, pH = 7), re-suspended, and filtered through glass wool. Conidia were stored in sterile PBS at −20°C. Conidia were stored no longer than six weeks prior to use based on in-house assessment of conidial viability under these conditions (unpublished data). *R. rhodochrous* strain DAP 96253 cells were maintained as glycerol stock aliquots (30% v/v) from 10 l fermentations carried out at GSU. Fresh glycerol stocks were used as the source of cells at the onset of each assay. The induction process was performed using the addition of urea or urea and cobalt as described in US patents 7,531,343 and 7,531,344 [8,10,11].

### Co-culture assays with *R. rhodochrous*

A single-compartment Petri plate (150 mm × 15 mm) was used for a contained air-space to assess *P. destructans* growth characteristics in the presence of induced cells of *R. rhodochrous*. A 10 μl inoculum of *P. destructans* conidia solution (10^6^ ml^−1^) in a phosphate buffer solution was spread onto SDA in Petri plates (35 mm x 10 mm). Multiply induced cells of *R. rhodochrous* [10,11] were inoculated onto Petri plates (35 mm × 10 mm) containing Yeast Extract/Malt Extract agar (YEMEA) with or without urea (7.5 g/l) [8], and cultured in the contained air-space for up to 30 days. All assays were conducted in triplicate. The ability of induced *R. rhodochrous* to inhibit healthy established hyphae of *P. destructans* was assessed using mycelial plug assays. A lawn of *P. destructans* was allowed to grow for up to 20 days at which time a 5-mm-diameter transfer tube was used to remove a plug from the mat of fungus. The plugs were then inserted into a similarly sized core removed from an uninoculated culture plate. The plates were co-incubated in a shared air-space as described previously and radial growth from the plug was assessed over time.

### Induced *R. rhodochrous* germule suppression assay

Thin layers (~750 μl) of 10% SDA were applied to standard microscope slides (24.5 × 76.2 mm) and 100 μl of *P. destructans* conidia solution (10^6^ ml^−1^) were spread across the agar surface. *R. rhodochrous-*inoculated Petri plates (35 mm × 10 mm) were placed in larger Petri plates (150 mm × 15 mm) and sealed with parafilm. Negative controls consisted of similarly-cultured conidia with no *R. rhodochrous* exposure. All trials were conducted in triplicate. At 4 and 7 days post-inoculation, conidia were observed in a light microscope at 200X magnification for the presence of germule formation. Germules were defined as single mycelial extensions emanating from conidia with a length equal to or greater than the intact conidia. Control and exposed slides were retained and examined daily for up to 21 days after germule formation was first observed on control slides. Recovery of conidia was determined by removing the *R. rhodochrous* after 24 hours, 72 hours, and 7 days. Slides were observed for 21 days after removal of control agent to assess recovery.

### Preparation and evaluation of fixed-cell catalyst and fermentation cell-paste in non-growth conditions

Immobilization of whole bacteria was carried out based on the methods of DeFilippi [12] and Lopez-Gallego *et al*. [13]. Refinement of immobilized cells to produce active catalyst was carried out according to the methods of Pierce *et al*. [10,11]. Evaluation of anti-*P. destructans* activity of fixed-cell catalyst and fermentation cell-paste was determined in co-culture assays with *P. destructans* conidia and mycelial plugs with various amounts of control agent (<1.0 g), as described previously. Efficacy was determined by observation of germule formation as compared to unexposed controls for growth from conidia, and as percent reduction in radial growth of mycelial plugs.

### *Ex vivo* anti-infectivity assay

The potential for induced *R. rhodochrous* to inhibit fungal growth on bat skin explants was evaluated using an *ex vivo* model of WNS. A 10-mm-diameter biopsy punch was used to collect full-thickness samples of skin (n = 40) from the patagium of bats (n = 2) immediately after euthanasia. The explants were adhered to a mesh support with tissue adhesive (TissueTek®) so that they would retain their shape and could be supported at the medium surface without allowing media to come in contact with the inoculated surface of the skin. The skin explants were then maintained on Eagle’s modified minimal essential medium supplemented with antibiotics (kanamycin, 100 μg/ml: amikacin, 20 μg/ml; and vancomycin 50 μg/ml). A suspension of spores was placed onto the center of the explant and allowed to dry. The inoculated explants were incubated in a shared air-space with induced *R. rhodochrous.* Uninoculated control explants were incubated alone or with uninduced *R. rhodochrous.* Initial experiments were conducted at 7°C. Anti-infective efficacy was determined by visual and microscopic evaluation of bat wing membrane tissue cultures exposed to induced *R. rhodochrous* as compared to unexposed and uninduced controls.

## Results

### Anti-*P. destructans* activity of induced *R. rhodochrous*

**Figure 1:**
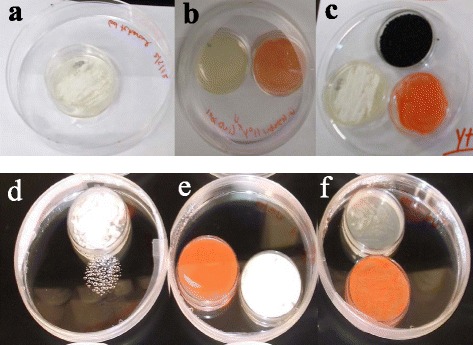
**Shared air-space co-culture of** ***P. destructans*** **conidia with** ***R. rhodochrous*** **.** Uninduced cells **(e)**, induced cells **(b, c and f)** and *P. destructans* control **(a, d)** were incubated in a shared air-space at 15°C (top panel) and 4°C (bottom panel). Induced *R. rhodochrous* fails to inhibit growth from conidia when activated carbon is included in the head-space **(c)**.

**Figure 2:**
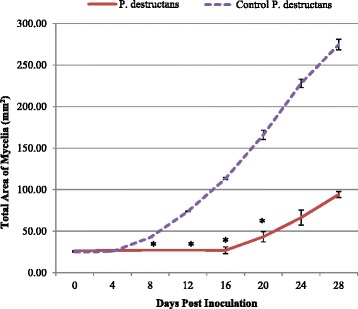
**Induced** ***R. rhodochrous*** **inhibits radial mycelial growth of** ***P. destructans*** **.** Growth areas of *P. destructans* plugs exposed to induced *R. rhodochrous* compared to *P. destructans* control plugs. All trials were conducted at 15°C. * indicates days post inoculation with statistically significant (P ≤ 0.05) radial growth inhibition.

Initial experiments with induced cells of *R. rhodochrous* demonstrated complete inhibition of growth from conidia of *P. destructans* when cultured with a shared air-space at 15°C (Figure 1a-c). Uninduced cells of *R. rhodochrous* showed no signs of inhibition, and were comparable to unexposed controls. Subsequent testing at 4°C demonstrated fungistatic activity of induced cells of *R. rhodochrous* and resulted in slower germination and reduced total mycelial growth as compared to uninduced cells of *R. rhodochrous* and unexposed controls (Figure 1d-f). Inclusion of activated carbon into the shared air-space abolished the anti-*P. destructans* activity of induced *R. rhodochrous* (Figure 1c). Mycelial plugs of *P. destructans* cultured in a shared air-space with induced *R. rhodochrous* had a significant reduction in radial mycelial extension as compared to control plugs cultured in the absence of induced cells of *R. rhodochrous* (Figure 2). Radial growth of induced *R. rhodochrous-*exposed *P. destructans* at 28 days post inoculation indicated a 35% reduction in radial mycelial extension as compared to unexposed controls. This inhibitory activity was statistically significant (p ≤ 0.05) on days 8, 12, 16, and 20 across all replicates (Figure 2).

### Induced *R. rhodochrous* permanently and persistently inhibits conidia germination

**Figure 3:**
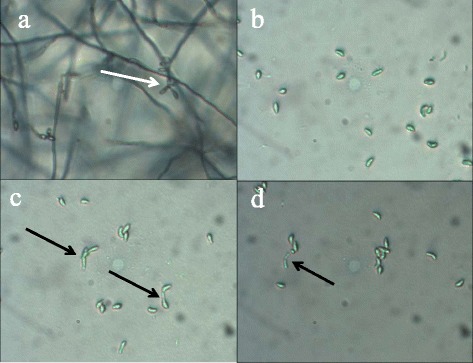
**Persistent suppression of** ***P. destructans*** **germination by induced** ***R. rhodochrous*** **.** *P. destructans* conidia are unable to recover after 24-hour exposure to induced *R. rhodochrous. P. destructans* control slide **(a)** produced significant mycelia growth and conidiation (white arrow) after 5 days*. P. destructans* conidia exposed to induced *Rhodococcus* for 24 hours **(b)**, 72 hours **(c)** and 7 days **(d)** failed to form germules 21 days after removal of induced *R. rhodochrous*. Halted germination was observed in 72-hour and 7-day exposures (black arrows). All images were captured at 200X magnification.

Slide agar overlays inoculated with *P. destructans* conidia and exposed to induced *R. rhodochrous* failed to produce germules 21 days after removal of *R. rhodochrous* (Figure 3). Conidia exposed to induced cells of *R. rhodochrous* for only 24 hours revealed no signs of germule formation, whereas conidia exposed for 4 and 7 days exhibited early signs of germination but no obvious germules (Figure 3).

### *Ex vivo* anti-infectivity activity of induced *R. rhodochrous*

**Figure 4:**
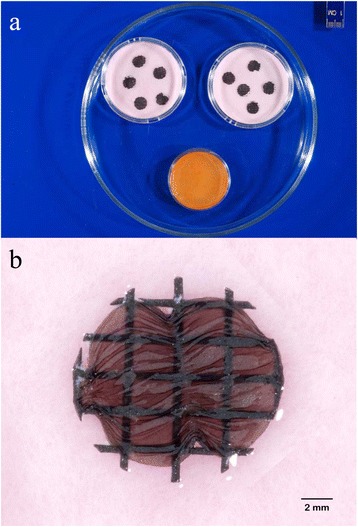
**Induced** ***R. rhodochrous*** **prevents fungal colonization of bat tissue when contained in a shared air-space.** Bat wing tissue explants in a shared air-space with induced *R. rhodochrous* 21 days post-inoculation with *P. destructans* conidia **(a)**. Magnified image of a control explant with visible fungal colonization 21 days post-inoculation **(b)**.

Induced *R. rhodochrous* completely inhibited the colonization of bat wing explants by *P. destructans* conidia in all replicates (n = 20) when incubated in a shared air-space for up to 21 days at 7°C (Figure 4). Explants exposed to uninduced *R. rhodochrous* and unexposed explants were fully colonized at 14 days post inoculation. Histopathological assessments of explants were conducted. However, in this experiment no fungal growth was detected on any induced *Rhodococcus* exposed explants. Therefore the histopathology of otherwise “healthy” explants provided no additional data to this experiment. Histopathology of the control explants adheres to the histopathology of WNS in bats as described by Cryan *et al*. [15]. Spore germination assays, and the bat wing explant study relied upon qualitative visual and microscopic evaluation and produced definitive results (i.e. no exposed explants developed fungal growth) therefore a statistical evaluation is unwarranted and omitted.

### Evaluation of fixed-cell catalyst and fermentation cell-paste

**Figure 5:**
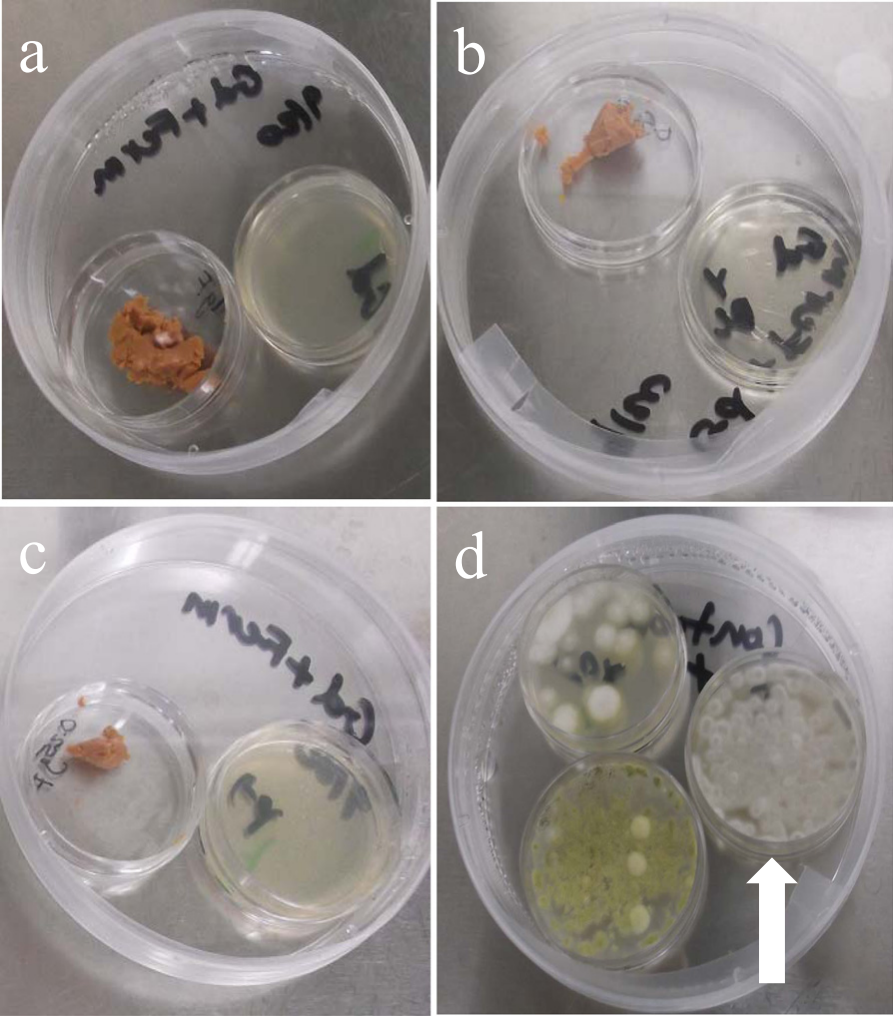
**Non-growth cell-paste of** ***R. rhodochrous*** **inhibits growth from conidia of** ***P. destructans*** **.** Non-growth fermentation cell-paste of induced *R. rhodochrous* was incubated in a shared air-space with *P. destructans* conidia inoculated plates. Quantities of 1.0 g, 0.5 g, and 0.25 g **(a, b, and c)** all demonstrated complete inhibition of growth from conidia of *P. destructans* as compared to unexposed controls (**d**, white arrow). Image taken 21 days post-inoculation.

Fixed-cell catalyst [8,10,11] failed to inhibit or slow growth from conidia of *P. destructans* when grown in a shared air-space. Fermentation cell-paste in quantities of 1.0 g, 0.5 g, and 0.25 g completely inhibited growth from conidia of *P. destructans* for greater than 80 days (Figure 5a-c).

## Discussion and conclusion

Since its initial documentation in 2006, WNS has spread to twenty-four states and four provinces and has been implicated in the mortality of millions of North American bats [16-18] which may have a significant impact on North American agricultural practices [19]. WNS is characterized by invasive mycelial growth on the wings, muzzle and ears of hibernating bats that perturbs physiological functions of the host tissues leading to mortality [15]. Cave closures and culling of infected individuals appears to have little to no impact on the spread and mortality associated with this devastating disease [20]. Classic disease management practices applied in agriculture, such as vaccination and broad-spectrum dissemination of antibiotics, present many challenges in the management of disease in wild, highly disseminated, and migratory animal populations. Consequently, the development of novel treatment options are needed to avert the spread of WNS and reduce the mortality associated with currently infected hibernacula. To this end, the development of biologically-based control tools is the preferred option for application in karst environments.

Since the publication of the national response plan [4], several groups have initiated investigations to identify potential biological control agents for *P. destructans* [21-23]. Several of the investigations have relied on traditional sources of biocontrol agents or probiotics such as bacilli and lactobacilli, or competitive exclusion fungi such as *Trichoderma* sp., as well as attempts to isolate bat-skin-associated microbes with anti-*P. destructans* activity [21-23]. While these approaches have proven successful in agricultural and human health applications [24-27], their application in the attempted remediation of WNS in bats has not been demonstrated. The requirement for contact with *P. destructans* and the bat hosts is a major hurdle for any agents reliant on competitive exclusion or non-volatile antimicrobial compound production. These potential control agents may prove to have limited efficacy against *P. destructans in situ* and potentially be harmful to the bat hosts. In contrast, the evaluation of induced *R. rhodochrous* strain DAP 96253 for application as a biological control agent of *P. destructans* aligns ideally with the needs of wildlife management agencies tasked with combatting WNS and is the first documented contact-independent microbial antagonism of *P. destructans*.

The evolutionary lineage of *R. rhodochrous* lends itself to VOC-based fungistasis due to its terrestrial ancestry [28-30]. The global prevalence of fungistatic soils is a measure of the natural antagonisms that exists in these complex environments [28-32]. Due to the ubiquity of *R. rhodochrous* in soils [5], it can be expected that *R. rhodochrous* as well as many other soil-dwelling bacteria have the potential to contribute to VOC-based fungistasis observed in these environments [29,30]. However, the development of induction methodologies is required to optimize this activity for biocontrol applications and is a decidedly advantageous quality of *R. rhodochrous* strain DAP 96253 as a potential biological control agent of WNS [33]. Leveraging this naturally evolved antagonism for control efforts has many benefits, particularly in the case of WNS. The complexity of soil ecology selects for antagonisms that are effective at low concentrations in diverse, compartmentalized environments where soluble diffusion may be limited [29]. Therefore, the production of antagonistic VOCs provides a viable means for soil-dwelling bacteria to compete with soil-dwelling fungi for resources and equates favorably with the environmental conditions of susceptible bat hibernacula. The ability of *R. rhodochrous* to detect and interfere with volatile signals has also been demonstrated in its delayed fruit-ripening activity [8] and is hypothesized to mediate the observed anti-*P. destructans* activity.

While the efficacy of urea-induced *R. rhodochrous* under growth conditions is promising for *in situ* management of WNS, the need for growth media supplementation poses problems for field application. The long term *in vitro* efficacy of non-growth-condition cell-paste at 4°C allows for increased confidence in forecasting the efficacy of this biocontrol agent in managing WNS in the field as this temperature is a sound approximation of average winter temperature of North American bat hibernacula [34]. The lack of growth media reduces the costs associated with application as well as reduces the likelihood of cross-contamination of control agent media with native cave microflora. In addition, the contact-independent basis of the non-growth antagonism will allow for *in situ* application methods that will reduce the potential for ecological impacts associated with introducing exogenous organisms to karst environments. The ecological impacts of any potential control agent are of significant concern for wildlife management agencies and the evaluation of potential ecological impacts must be assessed in order to circumvent ecological disasters associated with augmenting cave microflora (e.g. Lascaux cave) [4].

The evaluation of *R. rhodochrous* using *ex vivo* bat tissue explants as an indicator of anti-infective activity was paramount to establishing *R. rhodochrous* as a viable biocontrol agent of *P. destructans*. This was the first demonstration of inhibition of fungal colonization of bat tissue by a biological control agent. This *ex vivo* efficacy justifies further *in vivo* studies with live bats and should be pursued vigorously.

The ability of dormant conidia to remain viable in host-free environments increases long-term impacts of fungal pathogens and renders contaminated environments inhospitable to re-colonization [35]. The impact of WNS in locations such as New York has been tremendous, vastly reducing the populations of insectivorous bats over a broad geographic range. The permanent and persistent inhibition of conidia germination is a promising result and indicates that treatment of previously decimated hibernacula to inactivate resident conidia prior to re-colonization attempts may be feasible by applying induced *R. rhodochrous* in these environments. However further investigations are needed to confirm the applicability of this approach.

The evaluation of *R. rhodochrous* strain DAP 96253 has demonstrated the tremendous potential of this organism for application as a biological control agent of *P. destructans*. This is the first and only demonstration of contact-independent antagonism of *P. destructans* and represents a significant step toward the development of biologically-based treatment tools for WNS.

## References

[1] Lorch JM, Meteyer CU, Behr MJ, Boyles JG, Cryan JM, Hicks AC, Ballmann AE, Coleman JTH, Redell DN, Reeder DM, Blehert DS (2011). Experimental infection of bats with *Geomyces destructans* causes white-nose syndrome. Nature.

[2] Warnecke L, Turner JM, Bollinger TK, Lorch JM, Misra V, Cryan PM, Wibbelt G, Blehert DS, Willis CKR (2012). Inoculation of bats with European *Geomyces destructans* supports the novel pathogen hypothesis for the origin of white-nose syndrome. PNAS.

[3] Bastian F, Jurado V, Navakova A, Alabouvette C, Saiz-Jiminez C (2010). The microbiology of Lascaux cave. Microbiology.

[4] Ballman A, Benedict L, Britzke E, Castle K, Cottrell W, Cryan P, DeLiberto T, Elliot A, Ewing R, Hicks A, Reynolds R, Rubado J, Slack B, Williams L, Coleman J: **A national plan for assisting states, federal agencies, and tribes in managing white-nose syndrome in bats.** 2011. http://www.WhiteNoseSyndrome.org.

[5] Bell KS, Philp JC, Aw DWJ, Christofi N (1998). The genus *Rhodococcus*. J Appl Microbiol.

[6] Larkin MJ, Kulakov LA, Allen CC (2005). Biodegradation and *R. rhodochrous* –masters of catabolic versatility. Curr Opin Biotechnol.

[7] Nagasawa T, Shimizu H, Yamada H (1993). The superiority of the third-generation catalyst, *Rhodococcus rhodochrous* J1 nitrile hydratase, for industrial production of acrylamide. Appl Microbiol Biotechnol.

[8] Pierce GE, Drago GK, Ganguly S, Tucker T, Hooker JW, Jones S, Crow SA (2011). Preliminary report on a catalyst derived from induced cells of *Rhodococcus rhodochrous* DAP 96253 that delays the ripening of selected climacteric fruit: bananas, avocados, and peaches. J Ind Microbiol Biotechnol.

[9] Sunairi M, Iwabuchi N, Yoshizawa Y, Murooka H, Morisaki H, Nakajima M (1997). Cell-surface hydrophobicity and scum formation of *Rhodococcus rhodochrous* strains with different colonial morphologies. J Appl Microbiol.

[10] Pierce GE, Drago GK, Ganguly S: **Induction and stabilization of enzymatic activity in microorganisms.** 2009. US Patent 7,531,343.

[11] Pierce GE, Drago GK, Ganguly S: **Induction and stabilization of enzymatic activity in microorganisms.** 2009. US Patent 7,531,344.

[12] De Filippi LJ: **Process for preparing immobilized enzymes.** 1980. US Patent 4,229,536.

[13] Lopez-Gallaego F, Betancor L, Mateo C, Hidalgo A, Alonso-Morales N, Dellamora-Ortiz G, Gusian JM, Fernandez-Lafuente R (2005). Enzyme stabilization by gluteraldehyde crosslinking of absorbed proteins on aminated supports. J Biotechnol.

[14] Rajkumar SS, Li X, Rudd RJ, Okoniewski JC, Xu J, Chaturvedi S, Chaturvedi V (2011). Clonal genotype of *Geomyces destructans* among bats with White-Nose Syndrome, New York, USA. Emerg Infect Dis.

[15] Cryan PM, Meteyer CU, Blehert DS, Boyles JG (2010). Wing pathology of white-nose syndrome in bats suggests life-threatening disruption of physiology. BMC Biol.

[16] Blehert DS, Lorch JM, Ballman AE, Cryan PM, Meteyer CU: **Since 2007, infections by a previously unrecognized, perhaps imported fungus killed and estimated 1 million bats in North America.** *Microbe* 2011.

[17] Froschauer A, Coleman J: **North American bat death toll exceeds 5.5 million from white-nose syndrome.** 2012. U.S. Fish & Wildlife Service Press release.

[18] Gargas A, Trest MT, Christensen M, Volk TJ, Blehert DS (2009). *Geomyces destructans* sp. Nov. associated with white-nose syndrome. Mycotaxon.

[19] Boyles JG, Cryan PM, McCracken GF, Kunz TH (2011). Economic importance of bats in agriculture. Science.

[20] Hallam TG, McCracken GF (2011). Management of the panzootic white-nose syndrome through culling of bats. Conserv Biol.

[21] Amelon S, Knudsen G: **Identification and evaluation of potential biological control agents towards*****Geomyces destructans.*** *WNS Research Tracking-Draft* 2011. WNS Research Tracking- Draft.

[22] Chaturvedi V, Chatuvedi S: **Fungal biocontrol agents for alleviation or remediation of*****Geomyces destructans.*** Fiscal Year 2012 U.S. Fish and Wildlife Service-funded projects. FY 2012 USFWS-funded projects.

[23] Frick WF, Kilpatrick AM: **Antifungal skin microbes as tools for WNS management.** Fiscal Year 2012 U.S. Fish and Wildlife Service-funded projects. FY 2012 USFWS-funded projects.

[24] Berg G (2009). Plant-Microbe interactions promoting plant growth and health: perspectives for controlled use of microorganisms in agriculture. Appl Microbiol And Biotech.

[25] Pascual LM, Daniele MB, Ruiz F, Giordano W, Pajaro C, Barberis L (2008). *Lactobacillus rhamnosus* L60, a potential probiotic isolated from the human vagina. J Gen Appl Microbiol.

[26] Pitt JI, Hocking AD (2006). Mycotoxins in Australia: biocontrol of aflatoxin in peanuts. Mycopathologia.

[27] Wisniewski ME, Wilson CL (1992). Biological control of postharvest diseases of fruits and vegetables: recent advances. Hort Science.

[28] Chuankun X, Minghe M, Zhang L, Zhang K (2004). Soil volatile fungistasis and volatile fungistatic compounds. Soil Biol Biochem.

[29] Garbeva P, Hol WHG, Termorshuizen AJ, Kowalchuk GA, Boer WD (2001). Fungistasis and general soil biostasis – a new synthesis. Soil Biol Biochem.

[30] Kerr JR (1999). Bacterial inhibition of fungal growth and pathogenicity. Microb Ecol Health Dis.

[31] Ezra D, Strobel GA (2003). Effect of substrate on the bioactivity of volatile antimicrobials produced by *Muscodor albus*. Plant Sci.

[32] Fernando WG, Ramarathnam R, Krichnamoorthy AS, Savchuk SC (2005). Identification and use of potential bacterial organic antifungal volatiles in biocontrol. Soil Biol Biochem.

[33] Strobel GA, Kluck K, Hess WM, Sears J, Ezra D, Vargas PN (2007). *Muscodor albus* E-6, an endophyte of *Guazuma ulmifolia* making volatile antibiotics: isolation, characterization and experimental establishment in the host plant. Microbiology.

[34] Webb PI, Speakman JR, Racey PA (1996). How hot is a hibernaculum? A review of the temperatures at which bats hibernate. Can J Zool.

[35] Fisher MC, Henk DA, Briggs CJ, Brownstein JS, Madoff LC, McCraw SL, Gurr SJ (2012). Emerging fungal threats to animal, plant and ecosystem health. Nature.

